# All-Cause Mortality and Gastrointestinal Adverse Effects in Adults With Type 2 Diabetes on Glucagon-Like Peptide-1 Receptor Agonists vs Sodium–Glucose Cotransporter-2 Inhibitors

**DOI:** 10.1016/j.gastha.2025.100736

**Published:** 2025-07-05

**Authors:** Samita Garg, Thabet Qapaja, Osama Hamid, Gizem Kaya, Rashid Abdel-Razeq, Dina Alayan, Mohammed Abu-Rumaileh, Anthony Lembo, Steven Nissen

**Affiliations:** 1 Department of Gastroenterology, Hepatology and Nutrition, Digestive Disease Institute, Cleveland Clinic Foundation, Cleveland, Ohio; 2 Department of Hospital Medicine, Washington University in St. Louis, St. Louis, Missouri; 3 Department of Gastroenterology, University of Texas Southwestern, Dallas, Texas; 4 Department of Internal Medicine, Fairview Hospital, Cleveland, Ohio; 5 Department of Pulmonary and Critical Care, MetroHealth Medical Center, Cleveland, Ohio; 6 Department of Internal Medicine, University of Toledo, Toledo, Ohio; 7 Heart, Vascular and Thoracic Institute, Cleveland Clinic Foundation, Cleveland, Ohio

**Keywords:** GLP-1 receptor agonists, Type 2 diabetes, Gastrointestinal adverse effects, Gastroparesis, SGLT-2 inhibitor

## Abstract

**Background and Aims:**

Glucagon-like peptide-1 receptor agonists (GLP-1RA) are increasingly used in adults with type 2 diabetes (T2D), with or without obesity. The incidence of gastrointestinal (GI) adverse effects (AEs) of GLP-1RA in T2D is unclear. This study aimed to evaluate all-cause mortality and GI AEs in T2D patients treated with GLP-1RA compared to those treated with sodium–glucose cotransporter-2 inhibitors (SGLT-2i).

**Methods:**

A retrospective cohort study used electronic health records from the TriNetX Multi-Institutional Database (January 1, 2021, to December 31, 2022). T2D patients on GLP-1RA were compared to those on SGLT-2i. Primary outcomes were incident GI AEs and all-cause mortality. 1:1 propensity score matching was performed to reduce confounding, and adjusted odds ratios (aORs) with 95% confidence intervals (CIs) were calculated for each outcome.

**Results:**

The study included 3.2 million adults with T2D, with 104,947 prescribed a GLP-1RA compared with a matched cohort prescribed a SGLT-2i. After matching, baseline characteristics were similar, with a mean age of 62 ± 12 years and mean glycated hemoglobin of 8.0 ± 2.0%. About 30% of participants were from under-represented minority groups. At 24-month follow-up, patients prescribed a GLP-1RA had higher odds of being diagnosed with gastroparesis (GP) and gastroesophageal reflux disease (GERD) compared those prescribed a SGLT-2i, with aORs of 1.24 (95% CI, 1.11–1.38) for GP and 1.14 (95% CI, 1.11–1.18) for GERD. The risk of acute pancreatitis was lower in the GLP-1RA group. All-cause mortality at 24 months had an odds ratio of 0.83 (95% CI: 0.80, 0.87) compared to SGLT-2i.

**Conclusion:**

After 24 months of follow-up, patients treated with a GLP-1RA had higher odds of GP and GERD compared to those treated with a SGLT-2i. However, the odds of acute pancreatitis were lower in the GLP-1RA group. All-cause mortality was reduced by 17% in the GLP-1RA group.

## Background and Aims

Diabetes mellitus (DM) is a global health concern, affecting approximately 10.5% of adults worldwide, with projections indicating a significant increase to 12.2% by 2045.^1^ In the United States alone, an estimated 11.3% of the population (38.4 million people), have DM, primarily type 2 diabetes (T2D), which accounts for over 95% of DM cases.^2^, ^3^, ^4^, ^5^ Cardiovascular disease (CVD) and stroke are the leading causes of mortality among individuals with T2D. People with T2D have an increased CVD risk, with CVD occurring 2–4 times more frequently and resulting in a threefold increase in mortality compared to the general population.^6^

The GLP-1RA and dual-agonists (GLP-1RA + glucose dependent insulinotropic peptide) are recommended therapies for T2D, particularly in obese individuals associated with comorbidities. GLP-1RA stimulate glucose-dependent insulin release from pancreatic beta cells, suppress the paradoxical increase of glucagon after meals, and delay gastric emptying, thereby contributing to improved glycemic control and weight loss. These agents not only improve glycemic control but also confer cardiovascular (CVD) and renal (diabetic kidney disease) benefits as documented by several randomized controlled trials.^6^, ^7^, ^8^, ^9^, ^10^, ^11^ SGLT-2i are widely used in T2D as they also improve CVD and diabetic kidney disease outcomes^12^ but are not known to have significant gastrointestinal (GI) effects. Common AEs from SGLT-2i include metabolic and genitourinary issues. Hence we decided to compare the benefits and GI adverse effects (AEs) of these 2 classes of medications frequently prescribed in T2D.

Despite the CVD benefits associated with GLP-1RA, the comprehensive safety profile, particularly GI AEs remains incompletely characterized, especially in real-world populations.^13^, ^14^, ^15^, ^16^ Common GI AEs include gastroparesis (GP), gastroesophageal reflux disease (GERD), ileus, intestinal obstruction, pancreatitis, and biliary complications^13^^,^^17^, ^18^, ^19^, ^20^, ^21^, ^22^, ^23^, ^24^ though the risk of GLP-1RA in T2D in large studies may be different than in other conditions or smaller studies. The incidence and risk of these events may also vary based on population characteristics, drug formulations, and study designs, which are underexplored in large, diverse, real-world cohorts.^13^, ^14^, ^15^, ^16^ We evaluated all-cause mortality and GI AEs above in adults with T2D that were prescribed GLP-1RA or dual-agonists vs SGLT-2i over 2 years using a large multi-institutional deidentified database (TriNetX).

## Methods

### Database

We utilized deidentified electronic medical records from the large US Collaborative Network comprising 64 health-care organizations (HCOs) within the TriNetX Research Network, encompassing a total of 113 million patients. The dataset included demographic information, diagnoses (coded according to the International Classification of Diseases, Tenth Revision, ICD-10), procedures (coded in ICD-10-procedural coding system or Current Procedural Terminology), medication records, and laboratory tests (coded in LOINC). Additional ICD codes used in this data analysis are provided in the Appendix.

TriNetX is a health collaborative clinical research platform that gathers real-time Electronic Medtronic Records from various HCOs. TriNetX adheres to Health Insurance Portability & Accountability Act compliance and is exempt from institutional review board oversight as only deidentified data are used for analyses.

### Patient Selection

**Figure 1:**
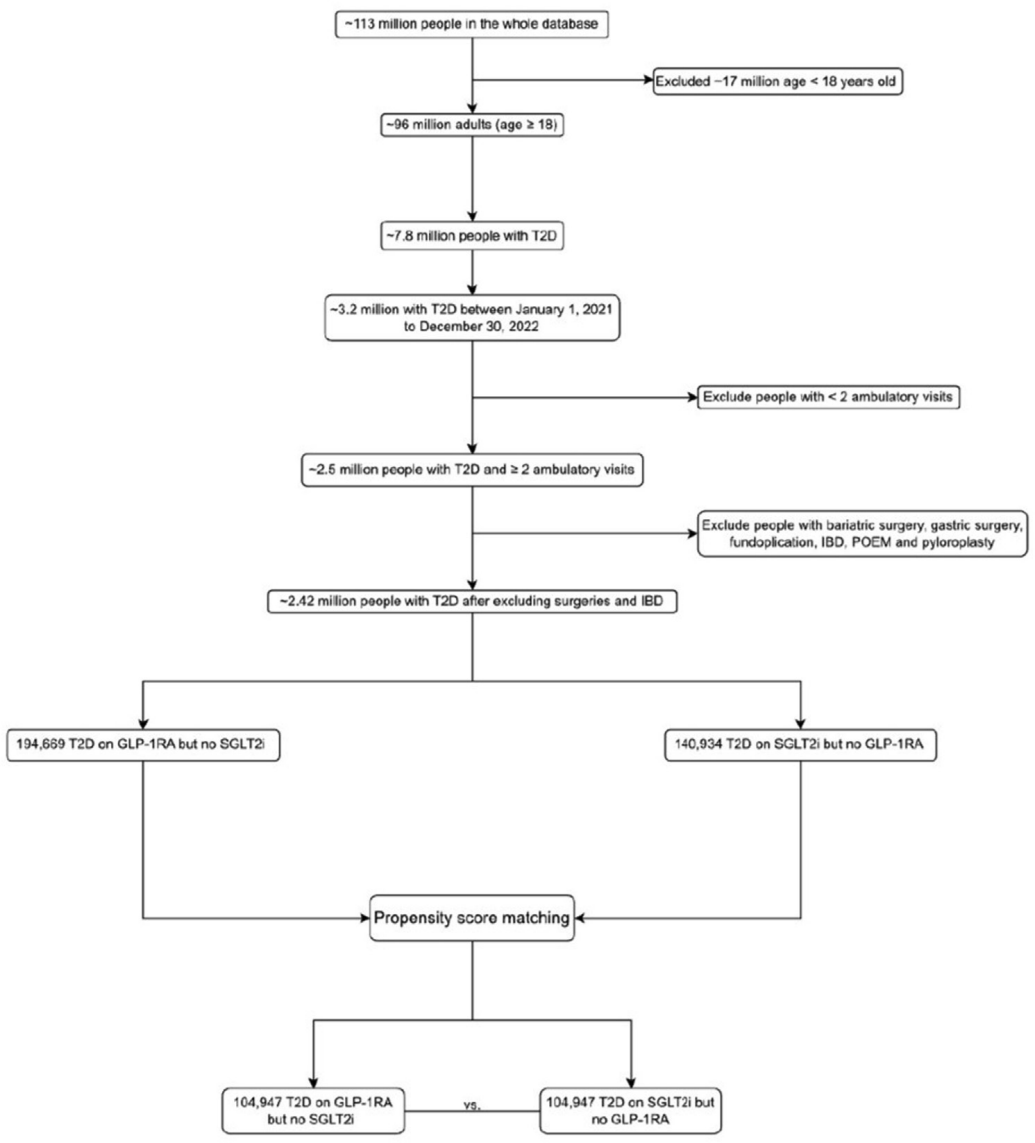
Patient selection flow diagram. The last 2 columns represent the final numbers of subjects in the 2 groups after propensity score matching. Patients with a history of bariatric surgery, gastric surgery, fundoplication, inflammatory bowel disease, per oral endoscopic myotomy, and pyloroplasty were excluded from the study. Patients with at least 2 ambulatory visits in the chart were included to ensure adequate follow-up.

Between January 1, 2021, and December 30, 2022, we identified a total of 3.2 million patients aged 18 years and older with a diagnosis of T2D (ICD-10 code E11). The cohort included 30% under-represented minorities (9% Hispanic, 16.8% African American, and 4.2% Asian). Patients were categorized into 2 groups before propensity matching^1^: Patients prescribed a GLP-1RA (dulaglutide, semaglutide, liraglutide, or tirzepatide) during the study period, with no concurrent prescription for an SGLT-2i, n = 194,669, and^2^ patients prescribed a SGLT-2i (dapagliflozin, empagliflozin, and canagliflozin) during the study period, with no concurrent prescription for a GLP-1RA, n = 140,934 (Figure 1).

After propensity matching there were 104,947 patients in both treatment groups (the GLP-1RA group and the SGLT-2i group) (Table 1 and Figure 1). We evaluated GI AEs such as GP, GERD, ileus, intestinal obstruction, acute pancreatitis, acute cholecystitis, cholelithiasis, and cholangitis at 3, 6, 12, and 24 months following the index date (defined as prescription of GLP-1RA and prescription of SGLT-2i). We also assessed all-cause mortality and incident major adverse cardiovascular events (MACEs), including myocardial infarction (MI), stroke, at 3, 6, 12, and 24 months following the index date. Patients with a prior history or diagnosis of these outcomes (GI AEs) at any time before the index date were excluded from the study.

**Table 1:** Baseline Characteristics of T2D on GLP-1RA vs SGLT-2i (After Propensity Score Matching)

	T2D + GLP-1RA N = 104,947	T2D + SGLT-2i N = 104,947	*P* value	Std Diff
Demographics				
Age at Index	62.0 ± 12.0	62.0 ± 12.0	.93	<0.001
White	63,428 (60.4%)	63,221 (60.2%)	.36	0.004
Female	44,427 (42.3%)	44,899 (42.8%)	.04	0.009
Unknown ethnicity	21,752 (20.7%)	21,557 (20.5%)	.29	0.005
Not Hispanic or Latino	73,712 (70.2%)	74,085 (70.6%)	.07	0.008
Hispanic or Latino	9483 (9.0%)	9305 (8.9%)	.17	0.006
Black or African American	17,591 (16.8%)	17,795 (17,795)	.23	0.005
Male	55,922 (53.3%)	55,395 (52.8%)	.02	0.01
Asian	4418 (4.2%)	4486 (4.3%)	.46	0.003
Comorbidities				
Parkinson disease	636 (0.6%)	642 (0.6%)	.87	0.001
Heart Failure	16,088 (15.3%)	16,477 (15.7%)	.02	0.01
Ischemic heart disease	28,155 (26.8%)	28,352 (27.0%)	.33	0.004
Chronic kidney disease	19,829 (18.9%)	19,944 (19.0%)	.52	0.003
Diseases of liver	14,828 (14.1%)	14,872 (14.2%)	.78	0.001
Alcohol-related disorders	3842 (3.7%)	3869 (3.7%)	.75	0.001
Nicotine dependence	15,235 (14.5%)	15,377 (14.7%)	.38	0.004
Constipation	12,388 (11.8%)	12,471 (11.9%)	.57	0.002
Polyneuropathies	9801 (9.3%)	9746 (9.3%)	.68	0.002
Neoplasms	33,642 (32.1%)	33,630 (32.0%)	.95	<0.001
Disorders of lipoprotein metabolism and other lipidemias	72,575 (69.2%)	73,044 (69.6%)	.03	0.01
Hypertensive disease	75,116 (71.6%)	75,562 (72.0%)	.03	0.009
Medications				
Opioids	52,651 (50.2%)	52,997 (50.5%)	.13	0.007
insulin	46,296 (44.1%)	46,383 (44.2%)	.7	0.002
Laxatives	44,666 (42.6%)	45,020 (42.9%)	.12	0.007
Proton pump inhibitors	39,242 (37.4%)	39,479 (37.6%)	.28	0.005
Aspirin	41,303 (39.4%)	41,778 (39.8%)	.03	0.009
Beta blocking agents	47,923 (45.7%)	48,277 (46.0%)	.12	0.007
Antilipidemic agents	72,752 (69.3%)	72,989 (69.5%)	.26	0.005
Angiotensin-converting enzyme inhibitors	45,081 (43.0%)	45,256 (43.1%)	.44	0.003
Angiotensin receptor blocker	32,498 (31.0%)	32,557 (31.0%)	.78	0.001
BMI/HbA1c				
BMI	34.1 ± 7.8	33.0 ± 7.3	<.001	0.149
HbA1c	8.0 ± 2.0	8.0 ± 1.9	<.001	0.021

Plus -minus values are means ± standard deviation. Numbers in parentheses are percentages. After propensity matching baseline characteristics were well balanced.

Patients with a history of bariatric surgery, gastric surgery, fundoplication, inflammatory bowel disease, per oral endoscopic myotomy, and pyloroplasty were excluded from the study since these diagnoses can affect gastrointestinal motility and may mask or amplify the effects of GLP-1RA. Patients with at least 2 ambulatory visits in the chart were included to ensure adequate follow-up.

### Statistical Analysis

TriNetX compares baseline characteristics using the chi-square test for categorical variables and the t-test for continuous variables. To mitigate the influence of confounding factors, we employed propensity score matching to create groups with similar baseline characteristics. We utilized TriNetX's built-in function to match the 2 groups at a 1:1 ratio through nearest-neighbor matching. The TriNetX platform uses input matrices with user-selected covariates to conduct logistic regression analysis, generating propensity scores for each individual subject. These propensity scores are then applied to match patients using a greedy nearest-neighbor algorithm with a caliper width of 0.1 pooled standard deviations. To minimize bias introduced by the order of data, TriNetX randomizes the row sequence before matching. Postmatching *P* values are provided to assess covariate balance between the 2 cohorts.

Propensity score matching was conducted for variables including age, sex, race, ethnicity, chronic kidney disease, hyperlipidemia, hypertension, liver disease, neoplasms, ischemic heart disease heart failure, alcohol-related disorders, nicotine dependence, constipation, Parkinson’s disease, neuropathy, and medications such as insulin, aspirin, beta blockers, antilipemic agents, angiotensin converting enzyme inhibitors, angiotensin receptor blockers, proton pump inhibitors, laxatives, opioids as well as laboratory values such as glycated hemoglobin (HbA1c), in addition to body mass index (BMI). Variables used for propensity score matching were obtained from any time before the index date. For continuous variables such as age, BMI, and HbA1c, the most recent value recorded before the index date was used.

Following propensity score matching, each outcome was evaluated and reported as an adjusted odds ratio (aOR) with a 95% confidence interval (CI). Statistical significance was defined as a 2-sided *P* value < .05. All analyses were conducted using the TriNetX Analytics function.

## Results

### Demographics

Following propensity score matching, minimal differences were observed in demographics, comorbidities, medications, and laboratory findings, indicating well-balanced matching (Table 1).

Patients prescribed a GLP-1RA had higher mean BMI compared to those prescribed a SGLT-2i (Table 2). The mean HbA1c remained elevated (>7.0%) in both groups. However, mean HbA1c tended to be lower at 24 months in patients prescribed a GLP-1RA treated group compared to patients prescribed a SGLT-2i (Table 2).

**Table 2:** Mean HbA1c and BMI in Patients With T2D on GLP-1RA vs SGLT-2i

Time since index date (mo)	Hemoglobin A1c (%)			BMI (kg/m^2^)		
	Mean HbA1c in T2D + GLP-1RA	Mean HbA1c in T2D + SGLT-2i	*P* value	Mean BMI in T2D + GLP-1RA	Mean BMI in T2D + SGLT-2i	*P* value
3	7.54 ± (SD 1.66)	7.65 ± (SD 1.60)	<.001	33.80 ± (SD 7.70)	32.33 ± (SD 7.24)	<.001
6	7.35 ± (SD 1.59)	7.53 ± (SD 1.52)	<.001	33.56 ± (SD 7.62)	32.18 ± (SD 7.13)	<.001
12	7.29 ± (SD 1.63)	7.49 ± (SD 1.54)	<.001	33.33 ± (SD 7.58)	32.04 ± (SD 7.10)	<.001
24	7.29 ± (SD 1.68)	7.51 ± (SD 1.58)	<.001	33.06 ± (SD 7.56)	31.88 ± (SD 7.10)	<.001

Plus -minus values are means ± standard deviation. BMI values are presented as kg/m^2^. Patients on GLP-1RA had higher mean BMI compared to the SGLT-2i group throughout the study duration. The mean HbA1c remained elevated (>7.0%) in both groups across all time points indicating suboptimal glucose control. However, mean HbA1c was lower at all times in the GLP-1RA treated group.

### GI AEs

Patients with T2D prescribed a GLP-1RA had a higher risk of receiving a diagnosis of GP and GERD at 24 months compared to patients prescribed a SGLT2i (aOR 1.24, 95% CI: 1.11–1.38 and aOR 1.14, 95% CI: 1.11–1.18, respectively). In contrast, the risk of ileus and acute pancreatitis was lower in patients prescribed a GLP-1RA compared to patients prescribed a SGLT2i (aOR 0.80, 95% CI: 0.70–0.91 and aOR 0.76, 95% CI: 0.67–0.85 respectively). No significant differences in the risk of acute cholecystitis, cholelithiasis, cholangitis or intestinal obstruction were observed at any time points (Table 3).

**Table 3:** Gastrointestinal Outcomes in Patients With T2D on GLP-1RA vs SGLT-2i (After Propensity Score Matching)

Time since index date (m)	T2D + GLP-1RA (n)	T2D + SGLT-2i (n)	aOR	95% CI
GP^a^				
3	145	138	1.05	(0.83–1.32)
6	266	227	1.17	(0.98–1.40)
12	447	400	1.12	(0.98–1.28)
24	723	583	1.24	(1.11–1.38)
Ileus				
3	91	131	0.69	(0.53–0.91)
6	143	202	0.71	(0.57–0.88)
12	259	326	0.79	(0.67–0.93)
24	400	502	0.80	(0.70–0.91)
GERD^a^				
3	2097	2247	0.96	(0.90–1.02)
6	3466	3465	1.03	(0.98–1.08)
12	5377	5142	1.08	(1.04–1.13)
24	7911	7210	1.14	(1.11–1.18)
Intestinal obstruction				
3	85	78	1.09	(0.80–1.48)
6	136	149	0.91	(0.72–1.15)
12	228	243	0.94	(0.78–1.12)
24	366	381	0.96	(0.83–1.11)
Acute pancreatitis				
3	98	165	0.58	(0.46–0.75)
6	166	267	0.61	(0.50–0.74)
12	309	420	0.72	(0.63–0.84)
24	496	645	0.76	(0.67–0.85)
Acute cholecystitis				
3	56	49	1.14	(0.78–1.68)
6	103	90	1.14	(0.86–1.52)
12	179	161	1.11	(0.90–1.38)
24	305	279	1.09	(0.93–1.29)
Cholelithiasis				
3	397	428	0.93	(0.81, 1.06)
6	662	680	0.97	(0.87, 1.08)
12	1153	1164	0.99	(0.91, 1.07)
24	1881	1886	1.00	(0.93, 1.06)
Cholangitis				
3	33	26	1.27	(0.76, 2.12)
6	54	46	1.17	(0.79, 1.74)
12	94	94	1.00	(0.75, 1.33)
24	137	139	0.99	(0.78, 1.25)

N = number of subjects with the respective event. 95% CI are provided for each column. AEs were selected based on the safety areas of interest for GLP-1RA.

a Only GP at 24 months and GERD at 12 and 24 months were significantly higher in those on GLP-1RA vs those treated with SGLT-2i. The risk of acute pancreatitis and ileus were lower at all time points in the GLP-1RA treated group. No significant differences in biliary outcomes or intestinal obstruction were observed at any time points.

### All-Cause Mortality and MACEs in the Treated and Control Groups

**Figure 2:**
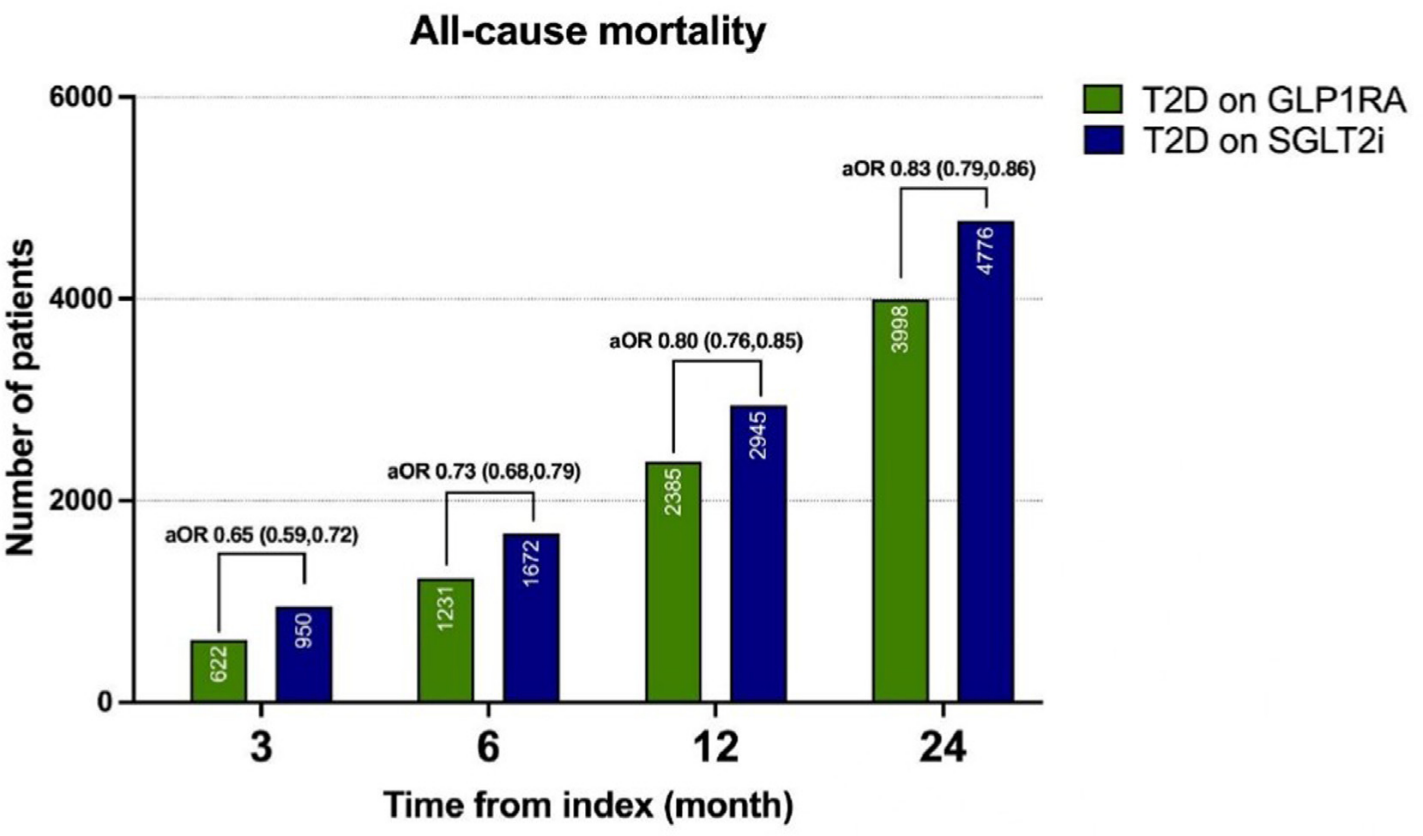
All-cause mortality in T2D on GLP-1RA vs SGLT-2i (after propensity score matching). All-cause mortality was 17% (aOR 0.83; 95% CI 0.79, 0.86) lower in the GLP-1RA treated group compared to SGLT-2i group at 2 years.

All-cause mortality rates were significantly lower in the GLP-1RA treated group at 3 months (OR 0.65, 95% CI: 0.59–0.72), 6 months (OR 0.74, 95% CI: 0.68–0.79), 12 months (OR 0.81, 95% CI: 0.76–0.85), and 24 months (OR 0.83, 95% CI: 0.80–0.87) when compared with patients on SGLT-2i (Table 4 and Figure 2). Similarly, patients prescribed a GLP-1RA had a reduced risk of MI and stroke at 3, 6, 12, and 24 months (Table 4).

**Table 4:** Mortality and MACE Outcomes in T2D on GLP-1RA vs SGLT-2i (After Propensity Score Matching)

Time since index date (mo)	T2D + GLP-1RA (n)	T2D + SGLT-2i (n)	aOR	95% CI
Mortality				
3	622	950	0.65	(0.59–0.72)
6	1231	1672	0.74	(0.68–0.79)
12	2385	2945	0.81	(0.76–0.85)
24	3998	4776	0.83	(0.80–0.87)
MI				
3	451	723	0.61	(0.54–0.69)
6	774	1141	0.66	(0.61–0.73)
12	1332	1819	0.71	(0.67–0.77)
24	2205	2719	0.79	(0.75–0.84)
Stroke				
3	470	664	0.70	(0.63–0.79)
6	793	1046	0.75	(0.69–0.83)
12	1324	1649	0.80	(0.74–0.86)
24	2114	2405	0.87	(0.82–0.93)

N = number of subjects with the respective event. 95% CI are provided for each column. All-cause mortality, MI, and Stroke were significantly lower in the GLP-1RA treated group throughout the 2-year period.

## Discussion

This real-world study, utilizing a large sample size, found that new diagnoses of GP and GERD were higher in patients prescribed a GLP-1RA compared to those prescribed a SGLT-2i. However, new diagnoses of acute pancreatitis and ileus were lower in the GLP-1RA group while new GI diagnoses such as cholecystitis, cholangitis, or intestinal obstruction were not significantly different. Furthermore, all-cause mortality, diagnosis of acute MI, and stroke were all lowered as early as 3 months of the initiation of GLP treatment and remained lower in the GLP-treated group at 2 years (*P* < .0001). We showed that all-cause mortality was lower by 17% in patients with T2D treated with GLP-1RA compared to SLGT-2i at 2 years despite having suboptimal glucose control and this is consistent with previous studies.^25^ Diagnoses of acute MI and stroke were reduced by 21% and 13% at 2 years respectively.

There are limited real-world data on the risk of GI AEs with GLP-1RA in T2D.^13^^,^^17^ We found an increased risk in new diagnosis of GP after 24 months of being prescribed a GLP-1RA and compared to patients prescribed a SGLT-2i. GLP-1RA is associated with delayed gastric emptying in contrast to SGLT-2i which have not been associated with delayed gastric emptying. Clinically we find GP occurs earlier but there may be a delay in diagnosis and gastric emptying testing may not have been performed right away though this specific information is not able to be ascertained from the database. In addition, there may be an effect of change in dosing or higher dosing that delayed gastric emptying. Furthermore, increased risk for new diagnosis of GERD in the GLP-1RA group occurred at 12 and 24 months compared to the SGLT-2i group. This may be partly related to an increased risk of GP. Increased risk of GERD in T2D taking GLP-1RA has been described previously with short-acting GLP-1RA.^18^ Our study included both short and long-acting GLP-1RA, latter which have been shown to demonstrate milder GI symptoms and greater glucose control.^26^

Studies assessing the risk of acute pancreatitis in adults with T2D on GLP-1RA have yielded conflicting results.^19^, ^20^, ^21^^,^^27^ A recent retrospective chart review of electronic health records at Cleveland Clinic did not show a higher risk of acute pancreatitis in patients with T2D taking GLP-1RA and a previous history of acute pancreatitis.^27^ Pooled analysis of cardiovascular outcome trials as well as systematic review of meta-analyses did not suggest increased risk of acute pancreatitis with GLP-1RA treatment in patients with T2D.^20^^,^^21^ A population-based cohort study reported an increased risk of hospitalization for acute pancreatitis with older short-acting GLP-1RA exenatide treated patients with T2D;^19^ however, the number of GLP-1RA users was small limiting statistical power to detect a difference. In contrast, our study, which included both short and long-acting GLP-1RA and larger sample size, did not identify a significant pancreatitis risk.

We also observed an insignificant risk of new diagnosis of biliary outcomes and intestinal obstruction. A nationwide cohort study reported liraglutide but not dulaglutide was associated with an elevated risk of biliary-related diseases compared to SGLT-2i in Asian patients with T2D^22^ but this risk was different in a diverse population with a heterogeneous array of GLP-1RA and predominantly long-acting GLP-1RA as in our study. Another systematic review and meta-analysis of randomized controlled trials found that use of GLP-1RA was associated with increased risk of gallbladder and biliary diseases but this was when GLP-1RA were used at higher doses and for longer durations for weight loss.^23^ When pooled with lower doses and shorter duration of GLP-1RA associated with less weight loss we found the risk of biliary disease was insignificant.

Lastly, in a large real-world study, GLP-1RA and dipeptidyl peptidase-4 inhibitors were associated with an increased risk of intestinal obstruction but this association was highest with 1.6–1.8 years of use.^24^ With shorter duration or lower dose of GLP-1RA, there was no significant increase in obstruction. Mohit et al found that use of GLP-1RA for weight loss (not T2D) compared with use of bupropion-naltrexone was associated with increased risk of pancreatitis, GP, and bowel obstruction but not biliary disease.^13^ The outcomes for T2D as in our study may be different than for weight loss, which is what the Mohit study evaluated.

The strengths of this study are (1) large database with patients from multiple institutions within the United States. (2) In addition, we analyzed multiple GLP-1RA analogs including semaglutide and tirzepatide over a 2-year period at multiple time intervals. (3) We had a diverse patient population with over 30% under-represented minorities. (4) Propensity matching for demographics, co-morbidities and medications that may have otherwise affected outcomes.

The limitations of this study include: (1) we could not confirm the diagnosis of GP with a gastric emptying study. (2) Patients may have been lost to follow-up due to various reasons, eg transitioning to HCOs outside of the TriNetX network. (3) This is a pooled analysis of all GLP-1RA, but it is possible that different GLP-1RA have different degrees of benefits or AEs. (4) It is not possible to know from the data alone whether patients actually filled the GLP-1RA prescriptions, treatment duration or dose, medication discontinuation, all of which may impact the interpretation of results. (5) We don’t know if the population was privately insured, self-pay or Medicare/Medicaid. (6) Approximately 26% of patients on SGLT-2i were not matched with GLP-1RA patients, which introduces potential selection bias and limits the external validity of the study.

## Conclusion

This is a large real-world study of GI AEs and all-cause mortality of GLP-1RA analog treatment for patients with T2D. Patients treated with GLP-1RA had increased risk of GP and GERD as well as decreased risk of MACEs and overall mortality events compared to those treated with SGLT-2i. The diabetes and gastroenterology communities, including regulatory agencies and payers, should consider these findings when assessing efficacy and AEs of these therapeutic agents. Greater awareness by both patients and providers can minimize and treat GI AEs associated with GLP-1RA use. However, future large prospective long-term studies are needed to substantiate our conclusions.
