# Supplementary material for: All-Cause Mortality and Gastrointestinal Adverse Effects in Adults With Type 2 Diabetes on Glucagon-Like Peptide-1 Receptor Agonists vs Sodium–Glucose Cotransporter-2 Inhibitors
Source: Gastro Hep Adv. 2025 Jul 5;4(10):100736. doi: 10.1016/j.gastha.2025.100736 (PMC12410549; doi:10.1016/j.gastha.2025.100736)
Supplement: Supplementary Material [file mmc1.docx]

**Appendix:**

| **Variable** | **ICD-10 code / CPT code / RXNORM code** |
| --- | --- |
| **Cohort creation** | |
| Type 2 diabetes mellitus | E11 |
| Dulaglutide | 1551291 |
| Tirzepatide | 2601723 |
| Liraglutide | 475968 |
| Exenatide | 60548 |
| Albiglutide | 1534763 |
| Lixisenatide | 1440051 |
| Semaglutide | 1991302 |
| Dapagliflozin | 1488564 |
| Empagliflozin | 1545653 |
| Canagliflozin | 1373458 |
| Laparoscopy, surgical, gastric restrictive procedure; longitudinal gastrectomy (i.e, sleeve gastrectomy) | 43775 |
| Gastric restrictive procedure, without gastric bypass, for morbid obesity; other than vertical-banded gastroplasty | 43843 |
| Laparoscopy, surgical, gastric restrictive procedure; with gastric bypass and small intestine reconstruction to limit absorption | 43645 |
| Gastric restrictive procedure, with gastric bypass for morbid obesity; with short limb (150 cm or less) Roux-en-Y gastroenterostomy | 43846 |
| Gastric restrictive procedure, with gastric bypass for morbid obesity; with small intestine reconstruction to limit absorption | 43847 |
| Gastric restrictive procedure, without gastric bypass, for morbid obesity; vertical-banded gastroplasty | 43842 |
| Unlisted laparoscopy procedure, stomach | 43659 |
| Restriction of Esophagogastric Junction, Percutaneous Endoscopic Approach | 0DV44ZZ |
| Esophagogastric fundoplasty, with fundic patch (Thal-Nissen procedure) | 43325 |
| Laparoscopy, surgical, repair of paraesophageal hernia, includes fundoplasty, when performed; without implantation of mesh | 43281 |
| Excision of Stomach, Open Approach | 0DB60ZZ |
| Bypass Stomach to Jejunum, Open Approach | 0D160ZA |
| Bypass Stomach to Jejunum, Percutaneous Endoscopic Approach | 0D164ZA |
| Excision of Stomach, Open Approach | 0DB60ZZ |
| Bypass Stomach to Jejunum, Open Approach | 0D160ZA |
| Bypass Stomach to Jejunum, Percutaneous Endoscopic Approach | 0D164ZA |
| Excision of Stomach, Open Approach | 0DB60ZZ |
| Bypass Stomach to Jejunum, Open Approach | 0D160ZA |
| Excision of Stomach, Percutaneous Endoscopic Approach, Vertical | 0DB64Z3 |
| Bypass Stomach to Jejunum with Nonautologous Tissue Substitute, Percutaneous Endoscopic Approach | 0D164KA |
| Bypass Stomach to Duodenum with Nonautologous Tissue Substitute, Percutaneous Endoscopic Approach | 0D164K9 |
| Bypass Stomach to Jejunum with Synthetic Substitute, Percutaneous Endoscopic Approach | 0D164JA |
| Bypass Stomach to Duodenum with Synthetic Substitute, Percutaneous Endoscopic Approach | 0D164J9 |
| Bypass Stomach to Jejunum with Autologous Tissue Substitute, Percutaneous Endoscopic Approach | 0D1647A |
| Bypass Stomach to Duodenum with Autologous Tissue Substitute, Percutaneous Endoscopic Approach | 0D16479 |
| Excision of Stomach, Open Approach | 0DB60ZZ |
| Bypass Duodenum to Duodenum, Open Approach | 0D190Z9 |
| Laparoscopy, surgical, gastric restrictive procedure; with gastric bypass and small intestine reconstruction to limit absorption | 43645 |
| Laparoscopy, surgical, gastric restrictive procedure; with gastric bypass and Roux-en-Y gastroenterostomy (roux limb 150 cm or less) | 43644 |
| Laparoscopy, surgical, esophagogastric fundoplasty (eg, Nissen, Toupet procedures) | 43280 |
| Lower esophageal myotomy, transoral (ie, peroral endoscopic myotomy [POEM]) | 43497 |
| Pyloroplasty | 43800 |
| Ulcerative colitis | K51 |
| Crohn's disease | K50 |
| **Outcomes** | |
| Acute myocardial infarction | I21 |
| Cerebral infarction | I63 |
| Gastroparesis | K31.84 |
| Paralytic ileus | K56.0, K56.7 |
| Gastro-esophageal reflux disease | K21 |
| Unspecified intestinal obstruction | K56.609 |
| Acute pancreatitis | K85 |
| Acute cholecystitis | K81.0 |
| Cholelithiasis | K80 |
| Cholangitis | K83.0 |
